# Systematic evaluation of adhesives for implant fixation in multimodal functional brain MRI

**DOI:** 10.1007/s10334-024-01220-4

**Published:** 2025-01-15

**Authors:** Anna Zsófia Szinyei, Bastian Maus, Jonas Q. Schmid, Matthias Klimek, Daniel Segelcke, Esther M. Pogatzki-Zahn, Bruno Pradier, Cornelius Faber

**Affiliations:** 1 Translational Research Imaging Center (TRIC), Clinic of Radiology, University of Münster, Albert-Schweitzer-Campus 1, building A16, 48149 Münster, Germany; 2 Department of Orthodontics, University of Münster, Albert-Schweitzer-Campus 1, building W30, 48149 Münster, Germany; 3 Department of Trauma, Hand and Reconstructive Surgery, University Hospital Münster, Albert-Schweitzer-Campus 1, building W1, 48149 Münster, Germany; 4 Department of Anesthesiology, Intensive Care and Pain Medicine, University Hospital Münster, Albert-Schweitzer-Campus 1, building A1, 48149 Münster, Germany

**Keywords:** Multimodal fMRI, Optogenetics, Mouse, Adhesives, Optical fiber

## Abstract

**Objective:**

Invasive multimodal fMRI in rodents is often compromised by susceptibility artifacts from adhesives used to secure cranial implants. We hypothesized that adhesive type, shape, and field strength significantly affect susceptibility artifacts, and systematically evaluated various adhesives.

**Materials and methods:**

Thirty-one adhesives were applied in constrained/unconstrained geometries and imaged with T2*-weighted EPI at 7.0 and 9.4 T to assess artifact depths. Spherical and flat patch shapes, both unconstrained geometries, were compared for artifact depth in vitro and in vivo. Adhesion strength was assessed on post-mortem mouse crania. Finally, an integrative scoring system rated adhesive properties, including artifact depth, handling, and adhesion strength.

**Results:**

Susceptibility artifacts were two times larger at 9.4 than at 7.0 T (*p* < 0.001), strongest at the patch edges, and deeper with spherical than flat patches (*p* < 0.05). Artifact size depended more on shape and volume after curing than adhesive type. Our integrative scoring system showed resins, bonding agents, and acrylics offered the best overall properties, while silicones and cements were less favorable.

**Discussion:**

Adhesive selection requires balancing handling, curing time, strength, and artifact depth. To minimize artifacts, adhesives should be applied in a spread-out, flat and thin layer. Our integrative scoring system supports classification of future classes of adhesives.

**Supplementary Information:**

The online version contains supplementary material available at 10.1007/s10334-024-01220-4.

## Introduction

Functional magnetic resonance imaging (fMRI) has become an essential tool in clinical and preclinical neuroscience, helping to explore and understand neural circuits and networks [1]. Among fMRI modalities, the blood oxygen level-dependent (BOLD) imaging is most commonly used [2]. The BOLD effect shows changes in blood flow, volume, and oxygenation due to neuronal activity, not direct neuronal firing. This limitation highlights the need to combine fMRI with techniques that measure neural electrochemical activity to better understand neuro-glial–vascular dynamics. In preclinical settings, various invasive methods are currently employed to achieve this. Fiberoptic-based methods, such as fiber photometry and optogenetics, are used to monitor or modulate neuronal activity. Fiber photometry uses fluorescence or bioluminescence and has been used with fMRI to detect real-time changes in ion concentrations, metabolites, and neurotransmitters [3–6]. Besides optical sensors, optogenetics uses light-sensitive ion channels or pumps to modulate neural activity, and has been frequently combined with fMRI (ofMRI) to examine how specific neural circuits affect brain-wide activity [7–9]. Additional techniques include direct neuronal recording or stimulation with specific electrodes [10–13], microdialysis for detecting neurotransmitters and metabolites [14], and drug infusion to modulate neural activity with pharmacological probes [15, 16]. Although relatively well established in rats, where larger brains and thicker crania reduce interference in imaging, these techniques still present challenges when combined with fMRI. These difficulties are exacerbated in mice, where thinner crania increase the risk of image artifacts caused by materials attached to the cranium. Despite these challenges, mice are increasingly favored in neuroscience research due to the availability of genetically modified mouse lines and disease models, and their smaller size.

Multimodal brain measurements in small rodents come with a high risk of fMRI image susceptibility artifacts from the implants, the trepanned cranium and the adhesives used to secure these implants [4, 17, 18]. Since tool implantation is necessary and the related artifacts cannot be avoided, minimizing susceptibility effects from the adhesives used for fixation is important. Hence, careful consideration of adhesives is crucial with respect to handling, adhesion properties, and minimizing susceptibility artifacts. Unfortunately, determining the composition of commercial adhesives beforehand can be challenging. Dental cements and acrylics are often employed due to their ease of use and biocompatibility [19, 20]. However, they frequently contain radiopaque additives and fillers, such as silica or metal oxides [21], which enhance X-ray contrast [22] and modify properties such as malleability and adhesive strength [23]. Despite these benefits, such additives may increase magnetic susceptibility [24], leading to severe image artifacts. Although such drawbacks are acknowledged in reference protocols [17, 19], the relationship between adhesive properties, such as handling, adhesive strength, and paramagnetic effects, remains largely unexplored.

To address this problem, we examined a total of 31 adhesives from multiple areas of use and chemical composition in vitro, ex vivo, and in vivo. In vitro studies evaluated the extent of image artifacts in T2*-weighted GE-EPI sequences, using a) standardized volumes and shapes and evaluated image artifacts at 7 T and 9.4 T (constrained geometry), followed by b) more realistic applications to account for viscosity and curing time (unconstrained geometry). The effects of different application shapes (flat vs. spherical) were also evaluated in vitro and in vivo for use in ofMRI. Selected adhesives were tested ex vivo for their adhesive strength on the mouse cranium. This systematic evaluation of adhesives provides insights into their suitability for multimodal fMRI in mice, particularly at high field strengths, and may offer guidance for their practical application in neuroimaging.

## Materials and methods

### Selection of adhesives

Adhesive selection was started with those referenced in fiber-based multimodal fMRI and neurophysiological studies [7, 17, 18, 25], as outlined in Online Supplement 1. Additional adhesives were included based on chemical class, availability, and affordability, and to introduce potentially new adhesive types for multimodal fMRI applications. Nail acrylics and household adhesives were specifically selected as accessible options to broaden the range of adhesives and to potentially introduce new adhesive types for multimodal fMRI. Clear nail acrylics, chosen for their lack of added pigments, and household glues, added due to their high accessibility, were included to address a wider variety of practical and experimental needs.

In total, we tested 31 adhesives for fiber fixation (Online Resource 1), grouping them based on conventional classifications commonly used in fields such as dentistry and orthodontics, as follows: silicone-based adhesives (sil.1–3), cement-based adhesives for medical use (cem.1–4), nail acrylics (nail.1–5), unfilled (unre.1–2) and filled dental resins (fire.1–6), bonding agents (boa.1–3), glass ionomer cements (glas.1), dental plasters (pla.1), and household acrylic adhesives (ha.1–5). A funnel approach was used to progressively eliminate adhesives with properties unfavorable to the experimental fMRI setting, with each experiment targeting specific properties. Therefore, not all adhesives were tested in every experiment.

For experiments with constrained geometry (see below), we employed 24 adhesives. For unconstrained geometry, we employed 30 adhesives. Shape tests and force measurements were performed on sil.2, glas.1, cem.1, nail.1, unre.1. The in vivo experiment used unre.1.

### Sample preparation for in vitro MRI

Three in vitro experiments were performed to assess susceptibility artifact formation and artifact depth caused by different adhesive properties. As sample, we used 20 mL syringes (diameter = 2 cm, length = 7.8 cm, BD Discardit™ II syringe, Becton Dickinson S.A., Fraga, Spain) filled with 1% agarose, because their curvature resembles a mouse cranium (hereafter referred to as tube). First, we evaluated the effects of magnetic field strength (7 T vs. 9.4 T) on susceptibility-induced signal deformations by applying adhesives in a standardized, constrained geometry at equal volume (see Sect. “Constrained geometry”). Second, to simulate practical application (e.g., on a mouse cranium), adhesives were applied unconstrained on tubes and examined at 9.4 T, accounting for differences in application techniques, handling characteristics (viscosity, precision of application, malleability), and curing (time and method, see Sect. "Unconstrained geometry"). Finally, to test the impact of patch shape, five adhesives were applied at equal weight (0.05 g) in two different shapes onto tubes (see Sect. "Adhesive shape comparison").

#### Constrained geometry

**Fig. 1:**
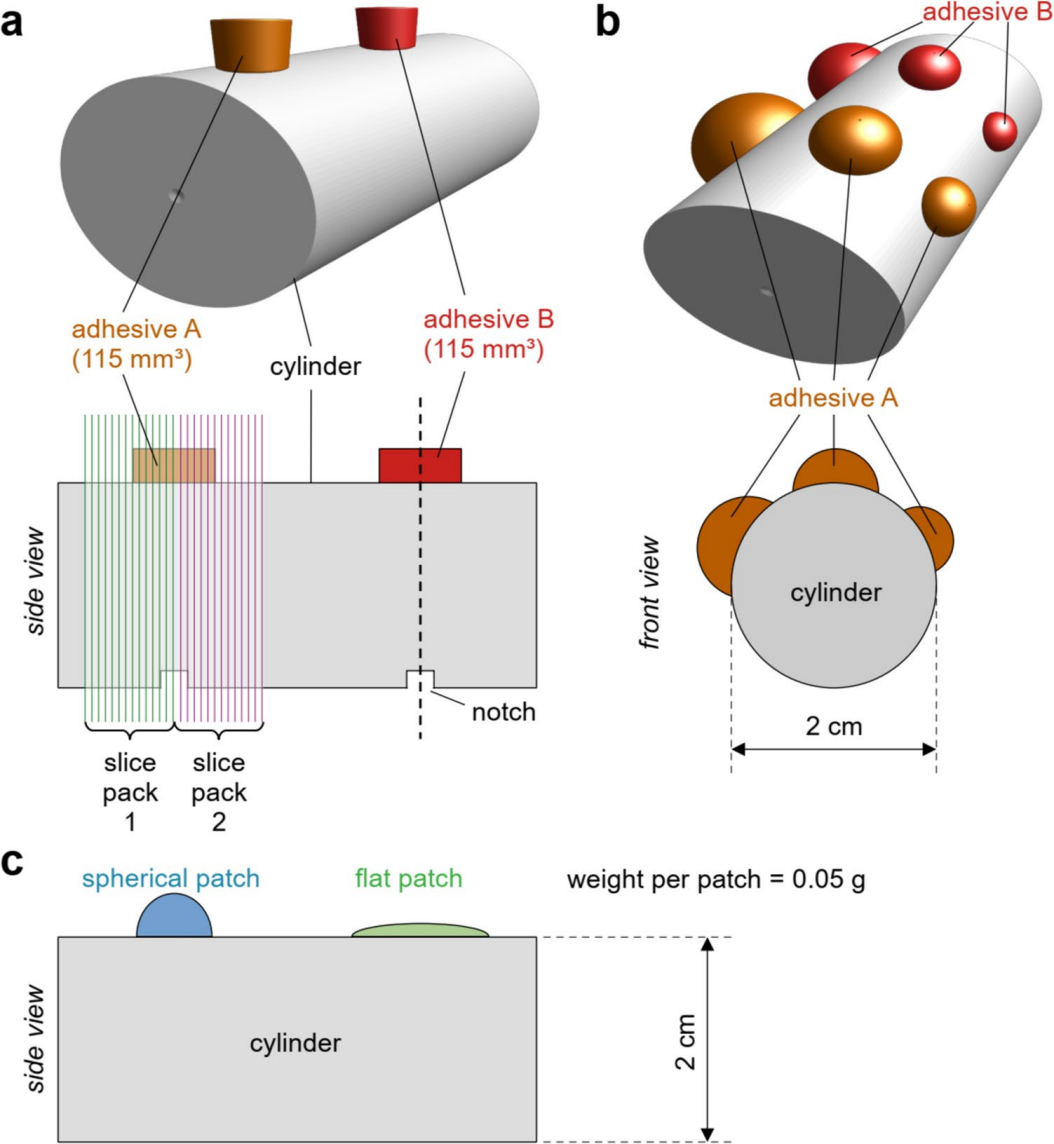
Adhesive-induced susceptibility artifacts were studied with different experimental setups. **a** 24 adhesives that were easy to handle were applied at defined shape and volume (115 mm^3^) on agar-filled tubes. The tubes were marked with notches opposite to the centers of the adhesive patches to facilitate alignment with the magnet center. Scans were acquired with two slice packages, aligned contiguously at the center of the notch and adhesive. **b** 30 adhesives were applied in triplet patches, and the corresponding artifact depths within the tube were quantified using plot profiles from axial cross sections (see 2.4.2). **c** Five adhesives were applied in different shapes (spherical vs. flat) at constant weight (0.05 g) per patch

To investigate the impact of magnetic field strength 7 T vs 9.4 T on susceptibility artifact formation, samples were prepared as follows: adhesives were applied in a defined cylindrical shape (*d* = 7 mm; *h* = 3 mm) and volume (115 mm^3^) to cylindrical tubes. A custom-made detachable Teflon mold was used to control the shape and volume of the adhesive patches, with the patches’ centers aligned to notches on the tubes to facilitate patch identification during MRI (Fig. 1a). The mold ensured that all adhesives were applied perpendicular onto the tube. The adhesive patches stayed in shape once the Teflon mold was removed. Only for boa.2, minor shrinking of the patch was observed.

#### Unconstrained geometry

Due to varying viscosities and handling characteristics, precise control over applied volumes was not possible, so we prioritized realistic application and natural dispersion to reflect actual use conditions. 30 adhesives were applied to agar-filled cylindrical tubes, with the amount determined by the adhesive’s viscosity to simulate practical use for fixing an experimental probe (e.g., optical fiber) to a mouse cranium.

All adhesives were applied to the horizontal tube with the applicator held at an angle. Application was usually performed in 4–6 small droplets, free of visible air entrapment, allowing them to melt and spread seamlessly. The droplets were shaped into three separate patches with varying amounts of adhesive, resulting in different heights and diameters (Fig. 1b). Patches with greater volumes exhibited a more spherical morphology, whereas smaller patches tended to be flatter. Low-viscosity adhesives were (light) cured before the subsequent application of droplets, whereas high-viscosity adhesives were applied without curing between drops.

For each adhesive, application techniques were optimized for the specific properties of each product, using either manufacturer-supplied applicators or adapted tools as needed (see below and Online Supplement 2), similar to established protocols [20]. Adhesives without built-in applicators were applied by syringe with a 20 G cannula (B. Braun SE, Melsungen, Germany) to form patches. When insufficient adhesive volume was available for syringe use, small amounts were collected on the cannula tip or on a rounded plastic stick, depending on adhesion to the applicator. Nail acrylics were applied with a manufacturer-supplied brush, producing larger drops (> 2 mm) for patch shaping. If sufficient amount of adhesive is available, application with a syringe and 20 G cannula improves precision. For two-component adhesives, mixing followed manufacturer guidelines, avoiding air inclusion. For certain high-viscosity adhesives, such as ha.2, complete removal of air inclusions proved challenging, impacting its final rating in the scoring system.

#### Adhesive shape comparison

To specifically assess the impact of patch shape on the extent and depth of susceptibility artifact formation, further investigations were performed on selected adhesives previously used in ofMRI or neurophysiology experiments (sil.2, glas.1 [17, 26]), those supplied with a standardized application protocol (cem.1), and those easy to handle under experimental conditions (nail.1, unre.1). Adhesive patches (0.05 g each) were applied in two different shapes onto agar-filled tubes: One patch was spherical with a small diameter and the other patch was flatter with a relatively larger diameter (Fig. 1c). Adhesives were shaped and smoothed using the manufacturer-built applicator, the tip of a 20 G cannula or a plastic stick.

### MRI

#### In vitro

To study the impact of adhesive properties on the formation of susceptibility artifacts, all samples were scanned with a 9.4 T small animal scanner (BioSpec 94/20, BGA12S gradient system: *G*_max_ = 720 mT/m, slew rate = 5000 T/m/s, ParaVision 6.0.1, Bruker BioSpin, Ettlingen, Germany). Additionally, to investigate the effect of field strength on susceptibility artifacts, the samples with constrained geometry were also scanned with a 7.0 T small animal scanner (BioSpec 70/20, BGA12S: *G*_max_ = 663 mT/m, slew rate = 4360 T/m/s, ParaVision 360v3.2, Bruker BioSpin). On both scanners, quadrature volume resonators (Bruker BioSpin) were used. Following adjustments, the sample position at the center of each scanner was determined in tri-pilot scans (fast low-angle shot (FLASH): TE = 4 ms, TR = 100 ms, flip angle = 30°, FOV = 40 × 40 mm^2^, 128 × 128 matrix, 2 mm slice thickness). Adhesive patches (identified via notches, Fig. 1a) were positioned in the center of the respective magnet and a local shim adjustment was performed under every patch, based on a *B*_0_ map (mapshim, Bruker BioSpin; 50% linewidth < 10 Hz for a 5 × 5 × 5 mm^3^ spectroscopy voxel). Finally, the extent of signal deformations associated with the adhesive patches was visualized using a multislice 2D gradient-echo EPI (GE-EPI) sequence. The sequence parameters reflected standard fMRI conditions [27, 28] and were as follows: TE = 18 ms; TR = 1 s; flip angle = 60°; FOV = 35 × 35 mm^2^; 100 × 100 matrix; 0.5 mm slice thickness with partial Fourier transform = 1.15 in phase-encoding direction; bandwidth = 277,777.8 Hz.

The samples with constrained geometry were recorded with two contiguous slice packages with 23 slices each to cover the entire extent of the patch and susceptibility artifacts while staying within the defined TR. The slice packages were placed around the center of each adhesive patch (Fig. 1a). All other experiments were performed only at 9.4 T. Signal deformations were determined from the same 2D GE-EPI sequence, however, here, only one stack of 23 axial slices was recorded per adhesive. The effects of adhesive patch shape on image artifacts were determined from axial and sagittal image stacks.

#### In vivo

Finally, the impact of adhesives’ properties on susceptibility artifacts due to patch shape was assessed in vivo during ofMRI experiments. These experiments were performed with a total of *n* = 8 C57BL6/J mice of both sexes (25.5 g ± 4.8 g) under an established isoflurane/medetomidine anesthetic regimen [28]. All experiments complied with the German Tierschutzgesetz and were approved by the State Agency for Nature, Environment and Consumer Protection North Rhine-Westphalia (LANUV, 81–02.04.2018.A013).

To prepare the cranium, a 1 cm-diameter area was exposed and etched for 15 s with 37% phosphoric acid (DMG Chemisch-Pharmazeutische Fabrik GmbH, Hamburg, Germany) to optimize adhesion [17]. Following craniotomy, an optic fiber (diameter 200 µm) was positioned at the primary sensory hind limb cortex (from bregma, anterior–posterior: − 0.82 mm, left–right: + 0.15 mm) at a depth of 300 µm from the dura mater. The optic fiber was secured with adhesive unre.1, applied in one of two ways: by creating multiple < 1 mm-diameter adhesive drops, combined into high patches with minimal cranium surface contact (spherical patch, Fig. 1c), or by spreading the adhesive over the entire exposed cranium area (flat patch, Fig. 1c). During application, the adhesive was repeatedly cured with blue light to prevent uncontrolled spreading due to unre.1’s low viscosity. In vivo MRI was performed at 9.4 T using a receive-only 2 cm surface coil, with the optic fiber passing through the coil’s center. A thin layer of low melting 1% agarose gel (Agarose Standard, Carl Roth GmbH & Co. KG, Karlsruhe, Germany) was applied over the fixed fiber and the adhesive [18]. While the gel cooled, a 2 × 2 cm^2^ piece of laboratory film (Brand^®^ Seal-R-filmTM, Brand GmbH & Co. KG, Wertheim, Germany) was placed between the agar and surface coil to protect the coil.

A quadrature volume resonator was used for excitation. While pre-scan adjustments were identical to the in vitro scans, the in vivo single-shot GE-EPI sequence was optimized for the morphology of a mouse cranium: FOV = 14 × 12 mm^2^; 70 × 60 matrix without partial Fourier transform; bandwidth = 200 kHz [27, 28]. Furthermore, a T2-weighted fast spin-echo sequence was used to image brain anatomy without susceptibility effects (rapid acquisition with relaxation enhancement, RARE: TE = 50 ms; TR = 2000 ms; 4 averages; RARE factor = 8; FOV = 14 × 12 mm^2^; 140 × 120 matrix; slice thickness and position corresponding to GE-EPI).

### Analysis of susceptibility artifacts via signal deformation

#### Analysis of samples with constrained geometry

**Fig. 2:**
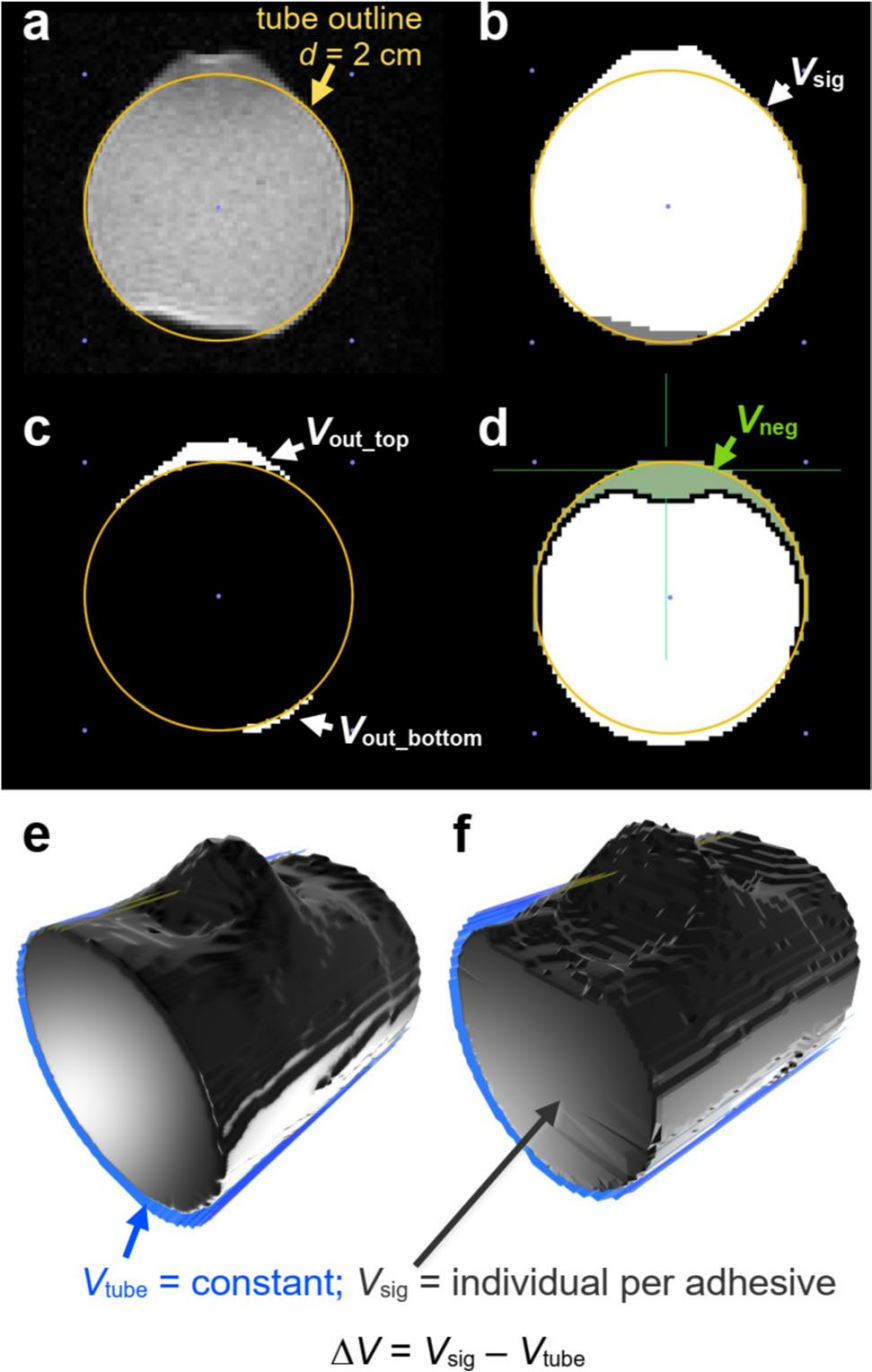
**a** At the center of an adhesive spot, the signal in an axial GE-EPI extended beyond a 2 cm-diameter circular ROI that represented the tube outline. The artifact at the bottom of the image derived from the notch underneath the adhesive (see Fig. 1a). **b** Segmentation of positive and negative signal deformations. *V*_sig_ thus included *V*_out_top_ and *V*_out_bottom_, but not the negative signal deformations *V*_neg_. **c** Positive signal deformations *V*_out_top_ and *V*_out_bottom_ protruded beyond the tube volume. **d** Negative signal deformations were found inside the tube volume. 3D renders of small **(e)** and large **(f)** volumetric deformations at 9.4 T associated with different adhesives at the top center of the volume

**Fig. 3:**
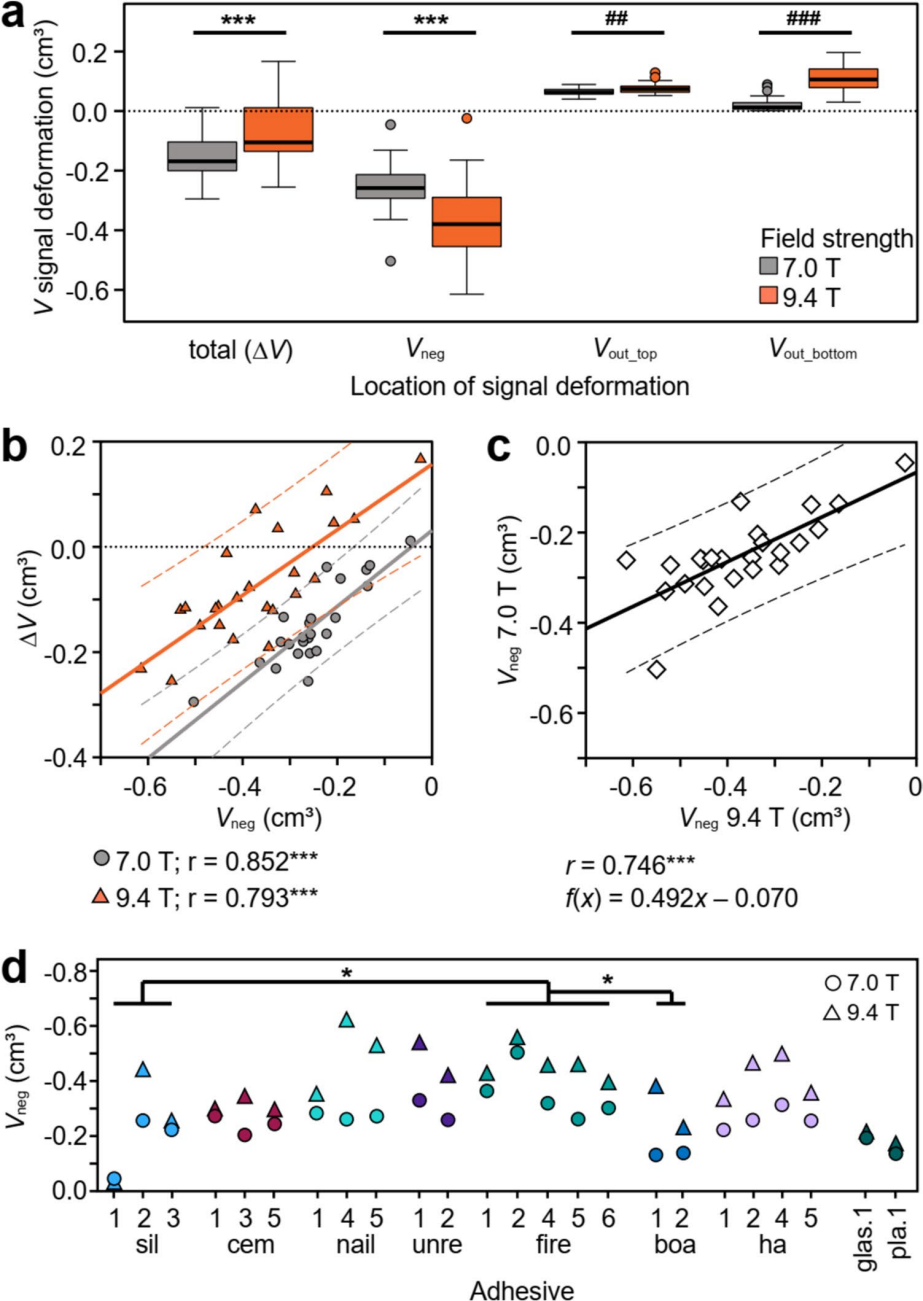
**a** Comparison of different volumetric signal deformations at 7.0 and 9.4 T. Boxes represent distributions for *n* = 24 adhesives. See Fig. 2 for location of the respective volumes. Central line = median, box = 0.5 percentile, whiskers 0.25 percentile, dots = outliers larger than 1.5-fold interquartile range. *** *p* < 0.001 (paired *t* test). ## *p* < 0.01 (Wilcoxon matched-pair signed-rank test). ### *p* < 0.001 (Wilcoxon matched-pair signed-rank test). **b** Linear relationship between Δ*V* and *V*_neg_. Pearson correlation coefficient *r* is significant for both regressions with *p* < 0.001. Solid lines: linear regressions. Dashed lines: respective 95% CI. **c** Linear relationship between *V*_neg_ at 7.0 and 9.4 T. Pearson correlation coefficient *r* is significant with *p* < 0.001. Solid line: linear regression. Dashed lines: 95% CI. **d** Comparison of *V*_neg_ for 7 T and 9.4 T. Different colors show different adhesive classes. Underscored classes were significantly different from each other at 7 T only (sil vs. fire and fire vs. boa, ANOVA, *p* < 0.05)

**Fig. 4:**
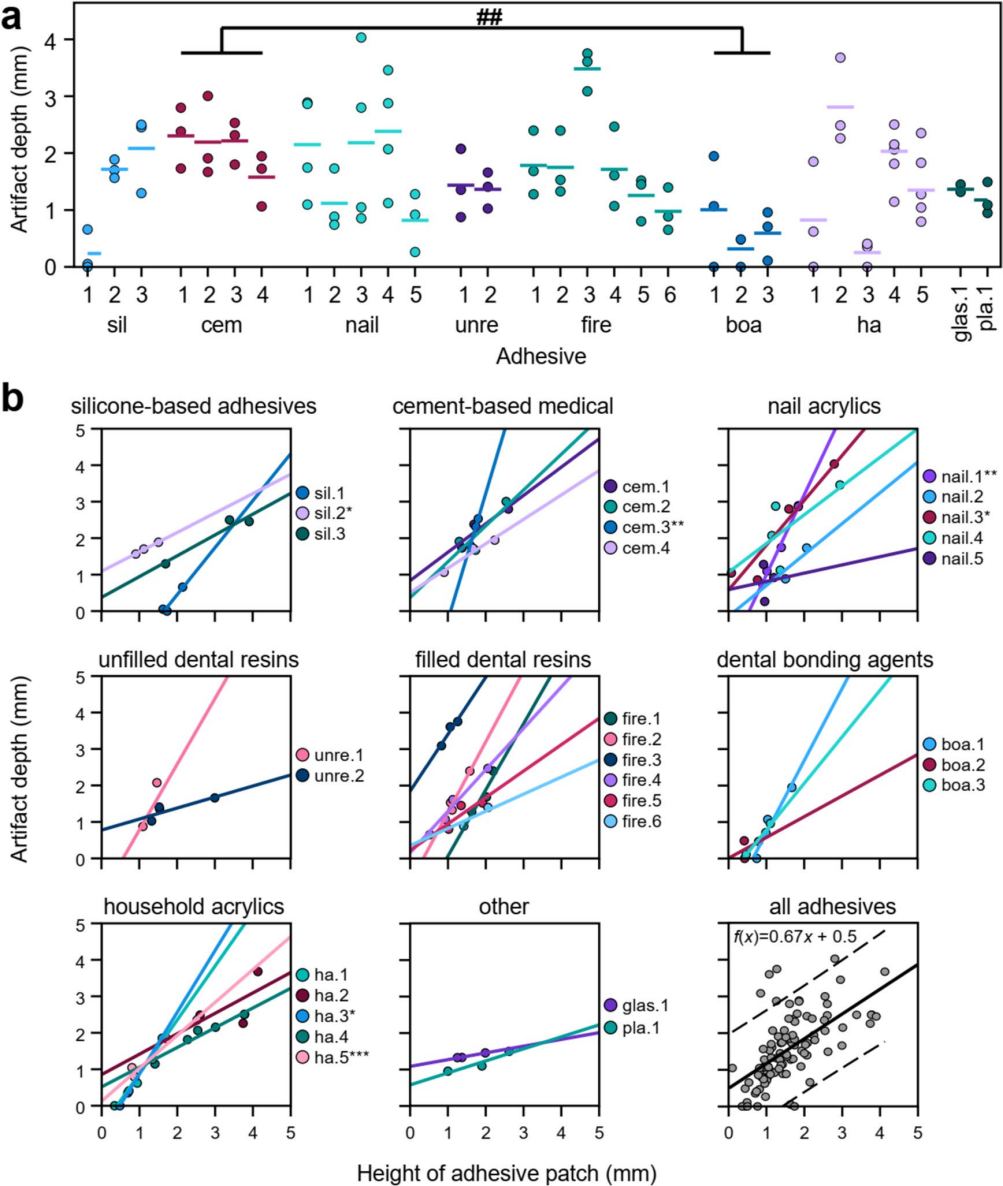
**a** Individual artifact depths for each patch of 30 adhesives following unconstrained application. Horizontal lines represent the mean per adhesive. A significant difference was only observed for classes cem. and boa. (Kruskal–Wallis test, ## *p* < 0.01). Note that some nail acrylics and household acrylics were more easily available and have thus been applied in more than three volumes. Sil—silicone based medical; cem—cement-based medical; nail—nail acrylic; unre—unfilled dental resins; fire—filled dental resins; boa—dental bonding agents; ha—household acrylics; glas—glass ionomer cement; pla—dental plaster. **b** Linear correlation between artifact depth and patch height. All correlations had Pearson’s *r* > 0.7, except for nail.4, nail.5, ha.2 and boa.2. Significant linear correlations are marked with asterisks (* *p* < 0.05; ** *p* < 0.01; *** *p* < 0.001). The linear correlation across all adhesives was significant (*r* = 0.615, *p* < 0.001); dashed lines represent 95% confidence interval

To analyze the effect of field strength on adhesive-induced susceptibility artifacts, we performed a volumetric analysis. For this purpose, the adjacent slice packages used to scan individual adhesive patches were stitched together using Horos 4.0.0 (Nimble Co LLC d/b/a Purview, Annapolis, MD, USA). First, the actual tube volume was fit to the stitched MR images via circular regions of interest (ROI) with a diameter of 2 cm per slice (total volume for 46 slices *V*_tube_ = 6.914 cm^3^, Fig. 2a). Then, a 3D growing region was placed to outline the deformed signal of the tube (*V*_sig_, Fig. 2b). The lower threshold for the growing region was one-third of the maximum signal intensity in slice 23 of the stitched slice package. From *V*_tube_ and *V*_sig_, the following volumetric signal deformations were determined: (1) total signal deformation Δ*V* = *V*_sig_–*V*_tube_ (Fig. 2e and f); (2) positive signal deformation beyond the top half of the syringe *V*_out_top_ (Fig. 2c); (3) positive signal deformation beyond the lower half of the tube *V*_out_bottom_ (Fig. 2c); (4) negative signal deformation inside the tube *V*_neg_ (Fig. 2d).

#### Analysis of samples with unconstrained geometry or defined adhesive shape

For in vivo experiments, the penetration of MRI signal extinction into the cortex poses a significant challenge for analyzing the BOLD signal, as critical neuronal structures, such as the pyramidal cell layers of the neocortex, lie 0.3–1.3 mm beneath the mouse cranium. Therefore, our subsequent analyses focused on characterizing the magnitude of susceptibility artifacts towards the edges of the adhesive patch. The artifact depth for each patch was quantified in vitro by measuring the maximum extent of the artifact. This was done by placing a line across the tube’s cross section, extracting the signal profile, and determining the half-maximum position from the fitted curve.

### Adhesive force measurements

In a preclinical MRI setting, the primary characteristic of adhesives, besides avoiding excessive artifact induction, is to securely attach implants to the cranium. Therefore, we measured their adhesive strength on the crania of post-mortem C57BL/6 mice for the adhesives that were also selected for shape tests (sil.2, glas.1, cem.1, nail.1, unre.1; see Sect. “Adhesive shape comparison”). The crania were prepared following a protocol similar to previous ofMRI experiments [17], with the surface etched for 15 s with 37% phosphoric acid (DMG Chemisch-Pharmazeutische Fabrik GmbH). Adhesives were applied within a 3 mm radius from Bregma with an unconstrained shape to secure a 4 mm loop of self-dissolving synthetic suture (Spool Suture PGA Violet, Henry Schein, Melville, NY, USA) to the cranium. The opposite end of the suture was attached to the materials testing machine (Zwick Z005, ZwickRoell GmbH & Co. KG, Ulm, Germany) through another loop. Mouse crania were clamped in the machine’s tensile direction aligned with the mechanical load on the cranium, adhesive, and suture. A 3D-printed clamping device, adapted for mice, was used to hold the cranium in place. A pull-out test was then performed. After a preload force of 1 N, the machine moved at 0.1 mm/s, recording data every 0.01 s. A force reduction to 80% of the maximum force was selected as the tear-out criterion and maximum force (*F*_max_) was recorded as quantitative measure. Adhesives were randomly tested on different crania, with glas.1 applied last since it was not possible to remove its residue. Each adhesive was tested on five crania, except sil.2, which failed to consistently adhere or sustain the pre-tensioning force, reducing the silicone samples from five to three.

### Integrative scoring of adhesive properties

To integrate the different characteristics we examined, we developed a scoring system to semi-quantitatively compare the properties of adhesives relevant in the context of fMRI. This system rates each characteristic from 0 (poor) to 3 (excellent; Table 1). Scores for image artifacts were based on a ranking of adhesives with lowest mean artifact depths from unconstrained geometry tests awarded highest scores. Handling was judged based on personal observations: it was best to fix fibers by applying multiple small adhesive drops (< 2 mm each) that can then be modeled into a flat patch around the fiber. Assisted curing using blue/UV light or compound mixing was preferential as it was faster than air curing. Insufficient handling was assigned to adhesives that lacked dedicated application methods, e.g., high viscosity prevented them from being applied via a syringe. Good handling was awarded to adhesives that came with a manufacturer-built applicator.

**Table 1:** Scoring system for adhesive properties

	Points	Criteria	Examples
Image artifact	3	Low	1.-8. lowest artifact extent
	2	Medium	9.-16. lowest artifact extent
	1	High	17.-24. lowest artifact extent
	0	> High	25.-30. (rank)
Handling	3	Medium/low viscosity AND patch shape can be easily controlled	Multiple drops with diameter < 2 mm may be easily applied with a manufacturer-built device
	2	Medium/high viscosity AND patch is difficult to shape precisely	Adhesive drop diameter 2–5 mm
	1	Low viscosity leads to uncontrolled spread over large area OR excessive deformation is part of curing process OR inefficient application	Undesired deformation of patch; no established (manufacturer-guided) application method
	0	Difficult to shape without using force OR lack of adhesion to bone	Adhesive sticks to the applicator rather than to the cranium
Curing	3	20–200 s	UV or blue light curing
	2	3–5 min	Mixing of two compounds to enable curing
	1	6–20 min	Air curing OR mixing of two components to enable curing
	0	> 20 min	Air curing
Strength	2	Peak adhesion *F*_max_ > 5 N	Maximum force applicable to the adhesive in the direction of the fiber (minimum of 3 repetitions per adhesive)
	1	Peak adhesion *F*_max_ 1–5 N	
	0	Peak adhesion *F*_max_ < 1 N	

Higher scores indicate better performance in the context of fMRI experiments

### Statistics

Pearson correlation coefficients were calculated to assess the linear relationship between artifact volumes at different field strengths and the relationship between susceptibility artifact depth and patch height after unconstrained application. All group results were tested for normal distribution (Shapiro–Wilk) and equal variance (Levene). For constrained geometries, results across adhesive classes were compared using one-way ANOVA with Tukey’s post hoc test. Positive and negative signal deformations between 9.4 T and 7.0 T were compared using paired *t* tests. In case of non-normal distribution or unequal variances, Wilcoxon matched-pair signed-rank tests were performed. To determine artifact depth for unconstrained geometries, a line was placed radially along the tube cross section to extract the signal profile using FIJI (ImageJ v1.53t); this profile was then fitted to a logistic function to determine the half-maximum position, using GraphPad Prism (v9, GraphPad Software, Boston, MA, USA). The height of an adhesive patch together with the associated artifact depth, as well as the maximum force results from the pull-out tests were compared across classes using the Kruskal–Wallis test (due to unequal variances) followed by an all-pairwise comparison with Bonferroni correction. Artifact depths of different adhesive patch shapes were compared using Student’s *t* test. All tests were performed with SPSS (v29, IBM, Armonk, NY, USA) at a significance level *α* = 0.05. Values are given as group means ± standard deviation.

## Results

First, in in vitro tests, we examined the effects of different field strengths and patch geometries on image artifacts induced by various adhesives. Next, we assessed *in *ex vivo testing adhesive strength on mouse crania. The findings helped develop a scoring system for assessing adhesives’ properties in rodent fMRI. Finally, the influence of adhesive patch shapes on artifact formation was evaluated in both in vitro and in vivo settings.

### Image artifacts for constrained geometry

When controlled for volume and shape, all adhesive patches on agar-filled tubes produced similar patterns of signal deformations, with the overall artifact structure consistent across both field strengths. A prominent conical positive signal deformation underneath the adhesive patch (*V*_out_top_) was observed, where the signal intensities of the agar-filled tube extended beyond the tube outline. This central positive deformation was always framed by two hypointense volumes towards both ends of the tube (*V*_neg_, Fig. 2e, f). The overall structure of the artifacts was identical at both field strengths. The hypointense volume at the lower half of the tube (*V*_out_bottom_) probably came from the central notch at the base of the tube, as its size was consistent. While it affected Δ*V*, it did not impact the adhesive-specific artifacts (*V*_neg_ and *V*_out_top_). Among these, *V*_neg_ exhibited larger depths across samples at higher field strengths (− 0.25 ± 0.09 cm^3^ at 7 T and − 0.37 ± 0.14 cm^3^ at 9.4 T), whereas *V*_out_top_ remained generally constant with ~ 0.07 cm^3^, approx. half the volume of the adhesive shape. Given the larger *V*_neg_ at constant *V*_out_top_, most adhesives caused a reduction of Δ*V* (Fig. 3a). Still, 6 of the 24 adhesives induced a net increase in Δ*V* at 9.4 T, compared to only one adhesive at 7.0 T because of strong positive deformations (Fig. 3b). Consequently, Δ*V* was significantly more positive at 9.4 T compared to 7.0 T despite the more negative *V*_neg_ at 9.4 T.

A significant positive linear relationship was found between Δ*V* and *V*_neg_ with Pearson correlation coefficients *r* > 0.79 for both field strengths. This highlights that *V*_neg_ is mainly responsible for the observed signal deformations. The slope of the linear regression is comparable between field strengths, though a distinction can be made in Fig. 3b, that reflects the more positive Δ*V* at 9.4 T.

The depths of hypointense artifacts *V*_neg_ were linearly correlated between field strengths (Fig. 3c). Across samples, the signal void at 7 T was ~ 50% smaller than the respective void at 9.4 T (Fig. 3a, c). Individual adhesives may differ from this relationship (Fig. 3d): silicone-based medical adhesives 1 and 3, cem.1, cem.5, nail.1, fire.2, glas.1 and pla.1 had similar respective *V*_neg_ at both field strengths, differing by < 20% for each adhesive. Of these, sil.1 had the lowest *V*_neg_ (0.02 cm^3^ at 9.4 T), followed by pla.1 (0.14 cm^3^, 7 T) and glas.1 (0.19 cm^3^). In contrast, nail.4 and boa.1 showed 2.5-fold larger *V*_neg_ at 9.4 T than at 7.0 T. Nail.4 had the largest *V*_neg_ at 9.4 T (0.61 cm^3^). Fire.2 had the largest *V*_neg_ at 7.0 T (0.50 cm^3^) and the second-largest *V*_neg_ at 9.4 T (− 0.55 cm^3^). Comparing adhesive classes, filled resins had the largest hypointense artifacts. At 7 T only, these were significantly larger than the artifacts generated by dental bonding agents and silicone-based adhesives. At 9.4 T, no significant differences were found across adhesive classes.

### Unconstrained geometry

Controlling adhesive shapes in an experimental setup is challenging due to viscosity, curing time, deformations, and the application method. This leads to arbitrary, unconstrained geometries. The artifacts induced by these unconstrained shapes are less uniform than those from constrained geometries. However, since the positive deformations depend on the amount of adhesive, we focused on the depth of negative image artifacts, which were analyzed by the maximal artifact penetration depth. Finally, we assigned scores to each adhesive based on unconstrained geometries, thus representing properties in real-world scenarios.

#### Image artifacts of unconstrained geometry application

While the principal structure of the image artifacts was consistent across adhesives with constrained geometries (see above), patches with unconstrained geometries induced a wide range of artifact depths, ranging from barely observable (mean depth < 0.4 mm: sil.1, boa.2, ha.3) to severe (mean depth > 2.5 mm, fire.3 and ha.2, Fig. 4a). The largest mean artifact depth was found for fire.3 (3.48 ± 0.35 mm), while other filled resins consistently showed artifact depths of ~ 1.5 mm. The artifact depths of dental bonding agents were significantly lower than those of artifacts associated with cement-based medical adhesives (Fig. 4a). This pattern was mirrored by patch height that was also significantly lower for dental bonding agents compared to cement-based and silicone-based medical adhesives. No further significant differences in artifact depth or patch height were found between adhesive classes.

The depth of artifacts was evidently affected by the quantity of adhesive applied. Typically, larger, more spherical patches resulted in the formation of larger negative artifacts. This is reflected in the overall positive linear correlation between patch height and artifact depth across adhesives (Fig. 4b). For sil.2, cem.3, nail.1, fire.1 and boa.1, the effect of patch height on artifact depth was particularly strong (Pearson’s *r* > 0.9). In contrast, nail.5, unre.2, glas.1, and pla.1 showed only a weak dependence on patch height. Across all samples, there was a significant positive linear correlation between patch height and artifact depth (*r* = 0.615, *p* < 0.001). Together, this shows that adhesive paramagnetic properties and the handling characteristics (i.e., ability to form small patches) both play important roles in artifact formation.

#### Adhesive strength

**Fig. 5:**
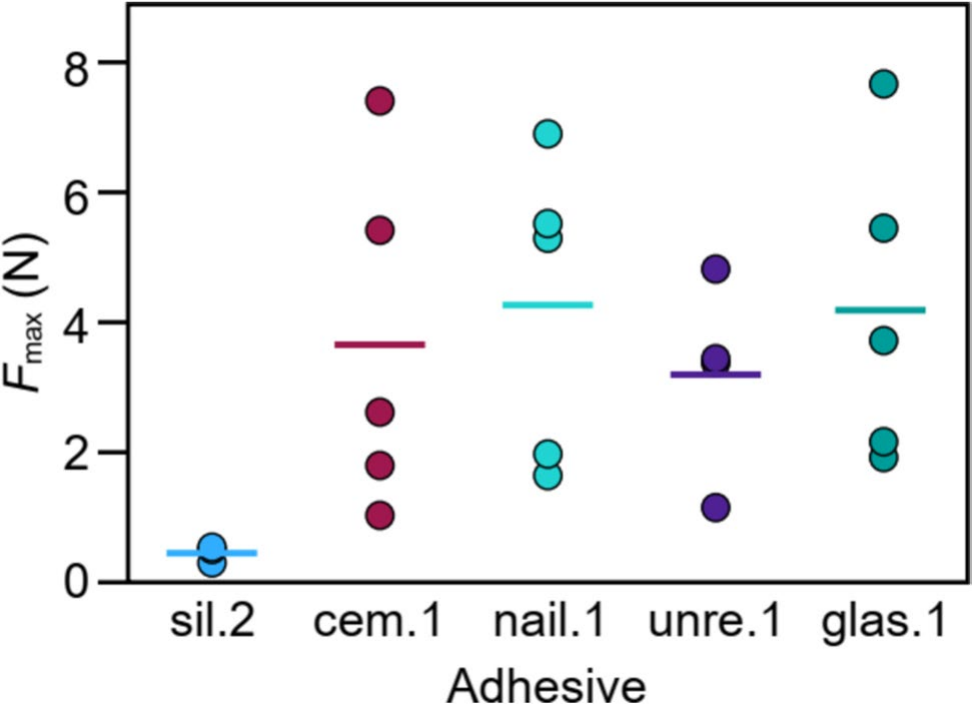
Maximum force *F*_max_ attained by five different adhesives during the pull-out test. Each data point reflects a successful pull-out test per adhesive. Central line = mean value. Kruskal–Wallis test revealed no significant differences

While the susceptibility effects of an adhesive are significant in MRI, its adhesive properties hold great interest. Therefore, a subset of adhesives was subjected to pull-out tests after attachment to an ex vivo mouse cranium in which the maximum adhesive force before pull-out was determined. On average, the nail acrylic (nail.1) withstood the highest *F*_max_ (4.3 ± 2.3 N), just slightly higher than the mean *F*_max_ of the glass ionomer cement (glas.1) at 4.2 ± 2.4 N. The absolute highest breakpoint force was also found for glas.1 at 7.67 N. Silicone-based adhesive 2 (sil.2) performed worst, withstanding only 0.5 ± 0.1 N (Fig. 5). For glas.1, pre-failure cracks occurred at varying force levels in three out of five trials. Cem.1 exhibited two initial pre-failure cracks at the start of testing in four out of five trials, while nail.1 showed two pre-failure cracks in one out of five trials. These pre-failure cracks were more frequent in the most brittle adhesives. Due to small replicate numbers and high data variance, no statistically significant differences were found. Still, the nail acrylic 1 withstood on average ten times the force of the silicone-based adhesive.

#### Scores

**Fig. 6:**
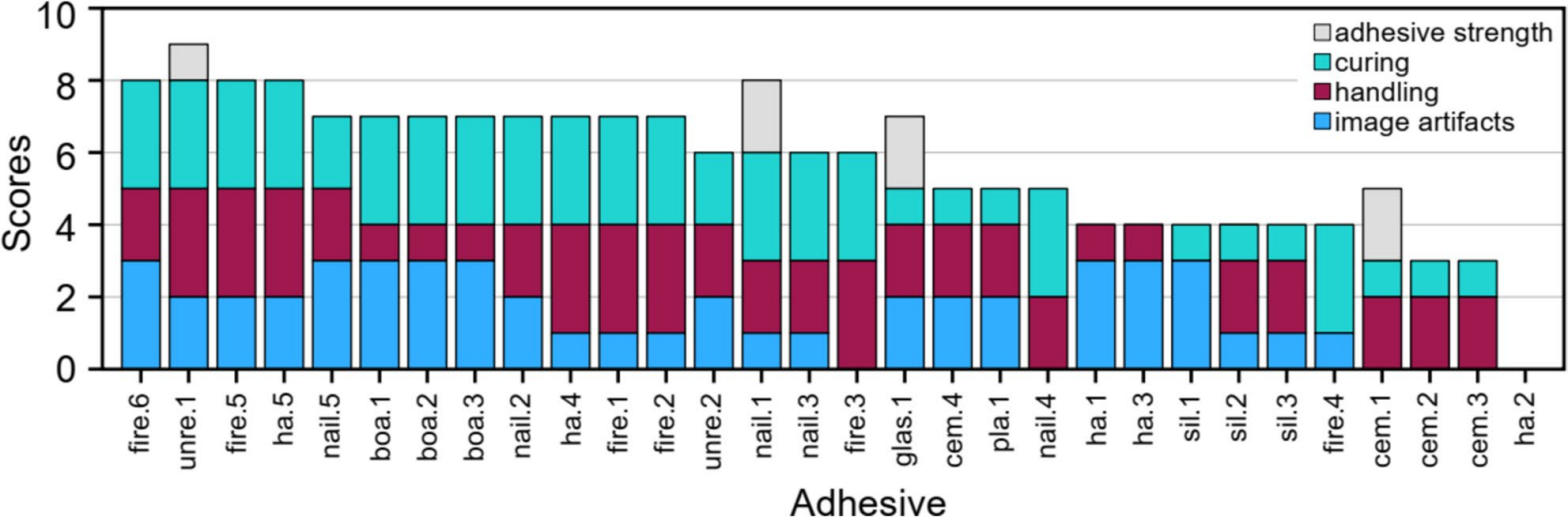
Summary of scores for (mean) artifact depth, handling, and curing. Higher scores indicate better performance in the respective category. Adhesive strength is additionally shown for those adhesives pull-out experiments were performed with: sil.2, cem.1, nail.1, unre.1, glas.1, and sil.2, with sil.2 scoring 0. Ha.2 scored 0 points in total. The adhesives were categorized based on their highest sum, excluding adhesive strength. This was followed by the highest score for image artifact depths, with the lowest depth being the preferred choice. Additionally, the sorting criteria included the best handling and best curing

No adhesive scored maximum points in the three categories (image artifact, handling, curing, sum = 9, best performance), though fire.6, unre.1, fire.5, and ha.5 each scored 8 points in total. (Fig. 6) All of them were exceptionally easy to cure and handle and induced only small image artifacts. Another eight adhesives scored 7 points in total. Here, a clear trend can be seen where top performance in one category comes at drawbacks in others: while nail.2, nail.5, and the dental bonding agents had top scores for image artifact depths, they were comparatively difficult to handle, scoring only 1–2 points in this category. Bonding agents had especially low viscosity. On the other hand, ha.4, fire.1, and fire.2 were much easier to handle (score 3), but incurred larger image artifact depths (score = 1).

23 adhesives scored 0–1 points in at least one category. This included all silicone-based and all cement-based adhesives. In case of cem.1, maximum adhesive strength (score = 2) was combined with difficult curing (score = 1) and large artifact depths (score = 0). Ha.2 scored 0 points in total (Fig. 6). More detailed information regarding the adhesives’ specific scores can be found in Online Supplement 2 (Fig. 6).

### Effects of adhesive shape in vitro and in vivo

**Fig. 7:**
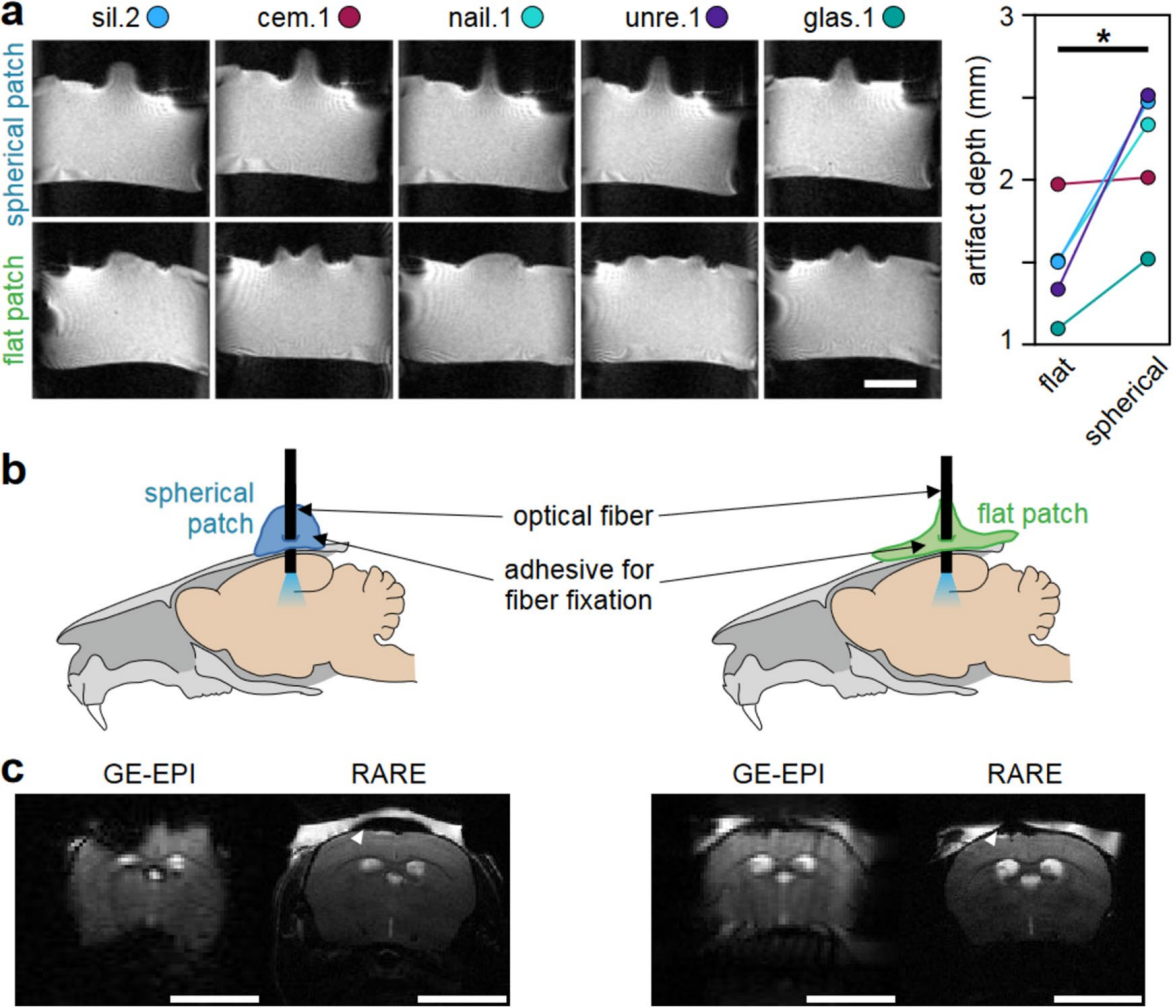
**a** Adhesive patches were applied to agar-filled tubes as spherical or flat patches (sagittal view, scale bar = 1 cm). Spherical patches had significantly larger artifact depths than flat patches (*p* < 0.05, *t* test). **b** Fixation of optical fiber on C57BL/6 mice crania using unre.1 as either spherical or flat patches. **c** Corresponding GE-EPI and T2-weighted RARE anatomy. Image position was 2–3 mm off the fiber insertion site. The extent of the adhesive patch is roughly delineated by the hypointense area (white arrow) in the RARE images, dorsal to the skull bone. Scale bar = 0.5 cm

Spherical patches caused signal deformations with a central hyperintense peak beyond the tube outline (*V*_out_top_), framed by hypointense areas 2–4 mm toward the ends of the tube (*V*_neg_). The overall shape of the artifacts appeared similar for all tested adhesives and was similar to those observed in the constrained geometry (Fig. 7a and Fig. 2e, f). Compared to spherical patches, the hyperintense deformations of flat, spread-out patches were shallower and extended further along the tube’s long-axis. Also, the hypointense areas were located further from the patch center (up to 1 cm). The central deformations of cem.1, unre.1, and glas.1 had a secondary artifact with reduced signal intensity close to the center of the flat adhesive patch. Every adhesive tested had larger depths for hypointense artifacts when applied in spherical patches, compared to flat patches. This difference was significant across all five adhesives (1.5 ± 0.3 mm for flat patches vs. 2.2 ± 0.4 mm for spherical patches, Fig. 7a). The impact of patch shape on artifact depth was largest for unre.1 and smallest for cem.1. Still, even for cem.1, flat, and spread-out patch shapes shifted the position of the (hypointense) artifact further away from the center of the patch.

In vitro results were confirmed during in vivo fMRI experiments: an adhesive applied as spherical patch induced relatively large artifacts in T2*-weighted GE-EPI, obscuring the mouse cortex. Again, the maximum depth of the artifact was not found underneath the insertion site, but 2–3 mm anterior and posterior, at the edge of the patch (Fig. 7b). Spread-out and flat application of adhesive on the cranium substantially reduced the artifacts in GE-EPI (Fig. 7c). The different shapes of the adhesive were easily identified as hypointense areas embedded in the hyperintense signal of agar placed on top of the cranium (Fig. 7b, c). Comparisons of artifacts associated with patch shape across different animals can be found in Online Supplement 3.

## Discussion

The present study revealed artifacts for all tested adhesives in T2*-weighted MR images. Their extent varied by type of adhesive and was larger at higher magnetic field strength *B*_0_. The adhesive-specific artifacts could not be attributed solely to the class of adhesive. Instead, the adhesives’ final shape and volume after curing had major impact on the position and extent of image artifacts.

The observed artifacts in the MR image result from differences in magnetic susceptibility at the interface of two substances [29, 30]. These interface geometries cause significant *B*_0_ inhomogeneities, which affect signal encoding [31], resulting in the observed image distortions. In vitro experiments with patches with constrained geometry revealed hypointense areas at the edges of all adhesive patches. These signal deformations are reminiscent to those occurring when cylinders are placed transverse to *B*_0_ [29, 30, 32]. This suggests that our volumetric analysis can be used to assess susceptibility effects. Hypointense areas at the edges of an adhesive patch were found for all 31 tested adhesives, some of which have been previously used for fMRI experiments and produced similar artifacts [17, 19]. While peripheral hypointense areas dominated, smaller hyperintense deformations underneath the patch center warped the underlying structures beyond their actual borders and may cause similar difficulties for image analysis. These artifact patterns were also observed when patch shapes were less standardized with unconstrained geometry. Differences between fast curing, viscous vs. slow curing, low-viscous adhesives led to variations in artifact shapes. Still, large artifact volumes for constrained geometries corresponded to large artifact depths for unconstrained geometries across adhesives. Another factor that had a major impact on the artifact formation was the field strength. As predicted by theory, signal voids at 9.4 T were twice as large as at 7.0 T. However, for certain adhesives, higher field strength may cause even larger image artifacts. While sil.2 has been successfully used in fMRI experiments at 7 T [17], our data suggest that at 9.4 T, susceptibility artifacts may obscure substantially larger parts of the cortex. The desired gains in fMRI sensitivity at higher fields may be offset by larger hypointense areas in the cortex.

Both patch geometry and magnetic field strength influence artifact formation, however, the adhesive's chemical composition also significantly contributes to variations in magnetic susceptibility. Notably, sil.1 and boa.2 had minimal susceptibility effects, regardless of shape, which suggests that the signal deformations observed in other adhesives were caused by their chemical composition, rather than by an arbitrary object placed on the syringe. (Metal) oxides such as SiO_2_, ZrO_2_ and Fe_2_O_3_ are commonly found in ceramics, added as fillers to dental adhesives. Further, oxides and reactive oxygen species are often formed during polymerization reactions as part of the curing process of acrylics. These molecular species are well known to increase magnetic susceptibility, since most of them are generally paramagnetic (with the exception of weakly diamagnetic SiO_2_). They nevertheless remain inherent components of most adhesives [21, 24, 29, 33, 34]. In this context, fire.5 and fire.6 induced relatively low susceptibility effects. They either did not contain sufficient amounts of susceptibility-inducing filler material, or the filler’s molecular configuration in the final compound caused only small magnetic susceptibility gradients to the agar [29]. All other filled resins and dental cements induced high susceptibility effects. However, it should be noted that an analysis of chemical composition and their individual contributions to magnetic susceptibility is beyond the scope of this work.

The different patch shapes with unconstrained geometry caused large within-group variations in susceptibility artifacts. The larger susceptibility effects observed in cement-based adhesives, in contrast to bonding agents, may be due to the spherical shape of the cement-based patches. However, the lack of significant differences between the two classes when using constrained geometries highlights the challenge of comparing standardized and practical application methods. For example, none of the class-dependent differences in artifact depth reported for constrained geometry were found when patch shapes were unconstrained. During unconstrained application, dental bonding agents could only be applied in flat patches because of the low viscosity of the compounds, highlighting the malleability as important adhesive property.

A high variance was also found for most adhesives during the force test. Our data show that silicone-based adhesives exhibit the weakest adhesion, while all other tested adhesives showed similar strong adhesion. Adhesion to the cranium is greatly improved with etching, but the degree of roughening depends on the experimenter and the individual differences in bone structure, such as those observed between female and male mice [35].

To achieve strong adhesion to the cranium, it is essential to remove the periosteum and any soft tissue from the cranium and to pad it dry. Adhesion can be promoted via chemical treatment [17, 36] or drilling screws as adhesion base into the cranium [37]. We decided to use phosphoric acid etching for its effectiveness to remove organic debris on dentin, dissolve hydroxyapatite, increase surface roughness, and to promote collagen bonding. To reduce the risks associated with prolonged etching, we recommend an etching time of 15 s [38]. However, since the cranium bone is highly vascularized and etching is not commonly used on bone, but on dental enamel, we further propose to explore alternative mechanical methods to increase retention such as burs, sandblasting, bone files or electric nail drills. If executed properly, these mechanical methods may offer effective and safer approaches for preparing the cranium for adhesive applications.

Multiple studies employed various adhesives for implant fixation [4, 7, 10] and recent protocols provide step-by-step instructions for conducting multimodal fMRI experiments [17, 19]. While these protocols provide guidance tailored to a specific experimental setup (e.g., specific implant materials or implant-to-cranium contact areas), they lack (systematic) comparison between different adhesives. To help researchers select the most appropriate adhesive for their specific needs, we developed a comprehensive scoring system, which integrates and evaluates key adhesive properties such as strength, curing time, handling, and image artifacts. By offering clear, property-based comparison, this system enables the selection of the most suitable adhesive for acute fMRI experiments, while easily eliminating ineffective options. For in vivo implant fixation, the ideal adhesives should combine strong adhesion with ease of handling, allowing for easy, flat application onto the cranium while still securely anchoring the implant. If the application surface is not horizontal, the adhesive patch shape must be carefully controlled by applying adhesive drops < 2 mm, with curing between applications to prevent flow due to gravity. Many household, silicone, and cement-based medical adhesives can be dismissed due to their long curing times (15–20 min) in relation to the open-skull animal surgery time (2 h). Ha.1 may only fully harden after 1 day, which is impossible to integrate in animal experiments. Additionally, cement-based adhesives often cause strong signal deformations, while household acrylics are difficult to handle and generally lack biocompatibility, they were included here mainly for comparison. Choosing an adhesive requires a strategic approach based on the experiments’ needs. Dental bonding agents, with low susceptibility artifacts and easy light curing, show promise but their low viscosity makes them difficult to mold into stable patches securing the implant on the cranium. However, when a thin adhesive layer is needed for secure bonding under an implant to the cranium, these may be the best option. Several adhesives ranked the highest in this study—all of them exhibiting excellent curing properties. The final choice depends on whether handling or minimizing artifact depth is to be prioritized. In our in vivo ofMRI experiments, precise handling was crucial, leading us to select unre.1 over fire.6, as a well-molded patch also reduced artifact depth. For cases requiring stronger adhesion, adhesives with longer curing times, such as with glas.1, may be acceptable. Finally, improving application methods could enhance the handling of adhesives like nail acrylics, making them more viable, affordable and accessible options for fMRI use.

This study provides a comprehensive overview of image artifacts induced by 31 adhesives, complemented by their application properties. As adhesives may find use in different MR scenarios, with specific demands, the presented scores offer guidance for choosing between particular adhesives. Depending on demand, high scores in individual characteristics can support choosing one adhesive over another if, e.g., adhesion or minimal susceptibilities are of primary importance and outweigh potential drawbacks. Constrained adhesive patch geometries revealed that image artifact depths were not universally linked to adhesive class. Taken together, the results indicate that any adhesive should be spread as largely, flatly, and thinly as possible to minimize artifacts and position them farthest from the area of interest.

## Supplementary Information

Below is the link to the electronic supplementary material.

Supplementary file1 (DOCX 696 kb)

## Data Availability

MR images (DICOM format) and raw data of the pull-out tests can be accessed in the “datastore” repository of the University Münster using the 10.17879/95968670828.
