# Supplementary material for: Systematic evaluation of adhesives for implant fixation in multimodal functional brain MRI
Source: MAGMA. 2025 Jan 15;38(2):191–205. doi: 10.1007/s10334-024-01220-4 (PMC11913989; doi:10.1007/s10334-024-01220-4)

# Online Supplements

**Online supplement 1** Summary and classification of adhesives. All adhesives were used for MR scans on unconstrained geometry

| **Class** | **ID** | **Trade name** | **Note** | **Constrained geometry** | **Force and shape tests** | **Literature reference** |
| --- | --- | --- | --- | --- | --- | --- |
| Silicone-based adhesives | sil.1 | S1 suhy, bisico®, Bielefeld, Germany |  | x |  |  |
|  | sil.2 | Kwik-Sil^TM^, World Precision Instruments, Sarasota, FL, USA |  | x | x | [17, 25, 39–41] |
|  | sil.3 | Silicon Rubber Compound, RS Components GmbH, Frankfurt am Main, Germany |  | x |  |  |
| Cement-based medical adhesives | cem.1 | Palacos® R+G – High viscosity, radiopaque bone cement containing Gentamicin, Heraeus Medical GmbH, Wehrheim, Germany |  | x | x |  |
|  | cem.2 | Palacos® R+G – High viscosity, radiopaque bone cement containing Gentamicin, Heraeus Medical GmbH, Wehrheim, Germany | The two components powder and liquid were mixed in a 1:1.5 ratio |  |  |  |
|  | cem.3 | Palacos® R+G – High viscosity, radiopaque bone cement containing Gentamicin, Heraeus Medical GmbH, Wehrheim, Germany  Unifast III, GC Corporation, Tokyo, Japan | A 1:1 ratio mixture of the powder-component of Palacos® with the liquid-component of Unifast following the instructions of Palacos® | x |  |  |
|  | cem.4 | Palacos® R+G – High viscosity, radiopaque bone cement containing Gentamicin, Heraeus Medical GmbH, Wehrheim, Germany  Unifast III, GC Corporation, Tokyo, Japan | A 1:1.5 ratio mixture of the powder-component of Palacos® with the liquid-component of Unifast following the instructions of Palacos® |  |  |  |
|  | cem.5 | Palacos® R+G – High viscosity, radiopaque bone cement containing Gentamicin, Heraeus Medical GmbH, Wehrheim, Germany  Unifast III, GC Corporation, Tokyo, Japan | A 1:1 ratio mixture of the powder-component of Unifast with the liquid-component of Palacos® following the instructions of Palacos® | Only used for constrained geometry, not for unconstrained geometry |  |  |
| Nail acrylics | nail.1 | Herbal Base, Hessi S.C., Koszalin, Poland |  | x | x |  |
|  | nail.2 | Take Your Time Builder, Indigo Nails Showroom Paris, Paris, France |  |  |  |  |
|  | nail.3 | Protein base removable, Indigo Nails, Łódź, Poland |  |  |  |  |
|  | nail.4 | Topdirect Polygel, Amazon Europe Core S.à r.l., Luxemburg, Luxemburg |  | x |  |  |
|  | nail.5 | Jolifin® Acryl Powder – clear, Jolifin®, Tangstedt, Germany  Nail Liquid, Indigo Nails, Łódź, Poland | Mixing and application following the instructions of Jolifin® | x |  |  |
| Unfilled dental resins | unre.1 | Helioseal® Clear, Ivoclar Vivadent GmbH, Ellwangen, Germany |  | x | x |  |
|  | unre.2 | Unifast III, GC Corporation, Tokyo, Japan |  | x |  | [42–46] |
| Filled dental resins | fire.1 | M+W Select Permaplast LH Viscous Flow, M+W Dental Müller & Weygandt GmbH, Büdingen, Germany |  | x |  | [17] |
|  | fire.2 | M+W Select Permaplast LH Flow, M+W Dental Müller & Weygandt GmbH, Büdingen, Germany |  | x |  | [17, 47] |
|  | fire.3 | 3M™ Filtek™ Universal Restorative A2, 3M Deutschland GmbH, Neuss, Germany |  |  |  |  |
|  | fire.4 | Blūgloo™, Ormco BV, Amersfoort, The Netherlands |  | x |  |  |
|  | fire.5 | ESTELITE UNIVERSAL FLOW, Tokuyama Dental Deutschland GmbH, Metelen, Germany |  | x |  |  |
|  | fire.6 | EQUIA Coat, GC Corporation, Tokyo, Japan |  | x |  |  |
| Dental bonding agents | boa.1 | OptiBond™ FL 2 Adhesive, Kerr Italia, S.r.l., Scafati, Italy |  | x |  | [17, 47] |
|  | boa.2 | iBond® Universal, Heraeus Kulzer GmbH, Hanau, Germany |  | x |  |  |
|  | boa.3 | MAXIMUM CURE® Part A/B, Reliance Orthodontic Products, Itasca, IL, USA |  |  |  |  |
| Glass ionomer cement | glas.1 | GlassLute™, PULPDENT®, Watertown, MA, USA |  | x | x | [26, 48–50] |
| Dental plaster | pla.1 | Moldano® Weiss, Kulzer GmbH, Hanau, Germany |  | x |  |  |
| Household acrylics | ha.1 | Acrifix 1R 0192 Acrylglaskleber, Röhm GmbH, Weiterstadt, Germany |  | x |  |  |
|  | ha.2 | Pattex® Alleskleber Stabilit Express, Pattex®, Henkel AG & Co. KGaA, Düsseldorf, Germany |  | x |  |  |
|  | ha.3 | Pattex® super glue liquid, Pattex®, Henkel AG & Co. KGaA, Düsseldorf. Germany |  |  |  |  |
|  | ha.4 | Pbond UV-ME 5140 thixo, Polytec PT GmbH Polymere Technologien, Waldbronn, Germany |  | x |  | [7, 18] |
|  | ha.5 | Pbond UV-PL 2010 FL, Polytec PT GmbH, Polymere Technologien, Waldbronn, Germany |  | x |  | [7, 18] |

**Additional references:**

39. Jung WB, Jiang H, Lee S, Kim S-G (2022) Dissection of brain-wide resting-state and functional somatosensory circuits by fMRI with optogenetic silencing. Proc Natl Acad Sci USA 119:e2113313119.

40. Tournissac M, Boido D, Omnès M, Goulam-Houssen Y, Ciobanu L, Charpak S (2022) Cranial window for longitudinal and multimodal imaging of the whole mouse cortex. Neurophoton. doi: 10.1117/1.NPh.9.3.031921

41. Mikkelsen SH, Wied B, Dashkovskyi V, Lindhardt TB, Hirschler L, Warnking JM, Barbier EL, Postnov D, Hansen B, Gutiérrez-Jiménez E (2022) Head holder and cranial window design for sequential magnetic resonance imaging and optical imaging in awake mice. Front Neurosci 16:926828.

42. Abe Y, Tsurugizawa T, Le Bihan D, Ciobanu L (2019) Spatial contribution of hippocampal BOLD activation in high-resolution fMRI. Sci Rep 9:3152.

43. Tsurugizawa T, Tamada K, Debacker C, Zalesky A, Takumi T (2021) Cranioplastic Surgery and Acclimation Training for Awake Mouse fMRI. BIO-PROTOCOL. doi: 10.21769/BioProtoc.3972

44. Abe Y, Sekino M, Terazono Y, Ohsaki H, Fukazawa Y, Sakai S, Yawo H, Hisatsune T (2012) Opto-fMRI analysis for exploring the neuronal connectivity of the hippocampal formation in rats. Neuroscience Research 74:248–255.

45. Tsurugizawa T, Yoshimaru D (2021) Impact of anesthesia on static and dynamic functional connectivity in mice. NeuroImage 241:118413.

46. Tsurugizawa T, Uematsu A, Uneyama H, Torii K (2010) Effects of isoflurane and alpha-chloralose anesthesia on BOLD fMRI responses to ingested l-glutamate in rats. Neuroscience 165:244–251.

47. Grimm C, Wenderoth N, Zerbi V (2022) An optimized protocol for assessing changes in mouse whole-brain activity using opto-fMRI. STAR Protocols 3:101761.

48. Lambers H, Wachsmuth L, Lippe C, Faber C (2023) The impact of vasomotion on analysis of rodent fMRI data. Front Neurosci 17:1064000.

49. Obermayer J, Luchicchi A, Heistek TS, De Kloet SF, Terra H, Bruinsma B, Mnie-Filali O, Kortleven C, Galakhova AA, Khalil AJ, Kroon T, Jonker AJ, De Haan R, Van De Berg WDJ, Goriounova NA, De Kock CPJ, Pattij T, Mansvelder HD (2019) Prefrontal cortical ChAT-VIP interneurons provide local excitation by cholinergic synaptic transmission and control attention. Nat Commun 10:5280.

50. Kohmann D, Lüttjohann A, Seidenbecher T, Coulon P, Pape H (2016) Short‐term depression of gap junctional coupling in reticular thalamic neurons of absence epileptic rats. The Journal of Physiology 594:5695–5710.

**Online supplement 2** Scoring table of adhesives used in unconstrained application for susceptibility, handling, and curing. Additional scores for adhesives based on strength testing

| **ID** | **Image artifact score** | **Mean artifact depth (mm)** | **Handling score** | **Handling notes** | **Curing score** | **Curing Notes** | **Overall Scores** | **Strength score** | **Overall Scores (incl. strength)** |
| --- | --- | --- | --- | --- | --- | --- | --- | --- | --- |
| fire.6 | 3 | 0.979 | 2 | relatively low viscosity; patch shape can be controlled; however, better applicator needed (capsule applier) | 3 | 20 s blue light (MEGALUX soft-start, Mega-PHYSIK GmbH & Co. KG, Rastatt, Germany) | **8** |  |  |
| unre.1 | 2 | 1.436 | 3 | low viscosity; manufacturer-built applicator allows precise application by creation of adhesive drops <2 mm | 3 | 20 s blue light | **8** | 1 | **9** |
| fire.5 | 2 | 1.257 | 3 | low viscosity; manufacturer-built applicator allows precise application by creation of adhesive drops <2 mm | 3 | 20 s blue light | **8** |  |  |
| ha.5 | 2 | 1.350 | 3 | medium viscosity; possibility to use in syringe with wide 20 G canula for application of adhesive drops <2 mm | 3 | 20 s UV light (LED flashlight with UV light, ENOVATEK GmbH, Jever, Germany) | **8** |  |  |
| nail.5 | 3 | 0.820 | 2 | medium viscosity; applicator brush limits creation of adhesive drops <2 mm | 2 | 3 min after combining the compounds | **7** |  |  |
| boa.1 | 3 | 1.005 | 1 | difficult to shape precisely due to low viscosity and an insufficient application method, as spreading cannot be controlled effectively | 3 | 20 s blue light | **7** |  |  |
| boa.2 | 3 | 0.316 | 1 | difficult to shape precisely due to low viscosity and an insufficient application method, as spreading cannot be controlled effectively | 3 | 20 s blue light | **7** |  |  |
| boa.3 | 3 | 0.592 | 1 | difficult to shape precisely due to very low viscosity and an insufficient application method, as spreading cannot be controlled effectively | 3 | 60 s after combining the compounds | **7** |  |  |
| nail.2 | 2 | 1.120 | 2 | medium viscosity; applicator brush limits creation of adhesive drops <2 mm | 3 | 40 s UV lamp | **7** |  |  |
| ha.4 | 1 | 2.031 | 3 | medium viscosity; possibility to use in syringe with wide 20 G canula for application of adhesive drops <2 mm | 3 | 20 s UV lamp | **7** |  |  |
| fire.1 | 1 | 1.787 | 3 | low viscosity; manufacturer-built applicator allows precise application by creation of adhesive drops <2 mm | 3 | 20 s blue light | **7** |  |  |
| fire.2 | 1 | 1.751 | 3 | medium viscosity; manufacturer-built applicator allows precise application by creation of adhesive drops <2 mm | 3 | 20 s blue light | **7** |  |  |
| unre.2 | 2 | 1.365 | 2 | medium viscosity; application by picking adhesive up with plastic stick tip limits creation of adhesive drops <2 mm | 2 | 5 min after combining the compounds | **6** |  |  |
| nail.1 | 1 | 2.149 | 2 | medium viscosity; applicator brush limits creation of adhesive drops <2 mm | 3 | 40 s UV lamp | **6** | 2 | **8** |
| nail.3 | 1 | 2.183 | 2 | medium viscosity; applicator brush limits creation of adhesive drops <2 mm | 3 | 40 s UV lamp | **6** |  |  |
| fire.3 | 0 | 3.483 | 3 | medium viscosity; manufacturer-built applicator allows precise application by creation of adhesive drops <2 mm | 3 | 20 s blue light | **6** |  |  |
| glas.1 | 2 | 1.364 | 2 | medium viscosity; application by picking up adhesive with tip of 20 G canula limits creation of adhesive drops <2 mm | 1 | 20 min after combining the compounds | **5** | 2 | **7** |
| cem.4 | 2 | 1.579 | 2 | medium viscosity; application with tip of 20 G cannula limits creation of adhesive drops <2 mm | 1 | 20 min after combining the compounds | **5** |  |  |
| pla.1 | 2 | 1.177 | 2 | medium viscosity; application by picking adhesive up with plastic stick tip limits creation of adhesive drops < 2 mm | 1 | 10 min after combining the compounds | **5** |  |  |
| nail.4 | 0 | 2.383 | 2 | medium viscosity; applicator brush limits creation of adhesive drops <2 mm | 3 | 40 s UV lamp | **5** |  |  |
| ha.1 | 3 | 0.824 | 1 | medium viscosity; difficult to shape precisely due to insufficient application method | 0 | 3 min UV lamp hardened it, needed a day in sunlight to fully cure | **4** |  |  |
| ha.3 | 3 | 0.253 | 1 | difficult to shape precisely due to very low viscosity and insufficient application method | 0 | air curing results in slow drying; the drying time is shortened when applied thinly between two objects | **4** |  |  |
| sil.1 | 3 | 0.237 | 0 | high viscosity; difficulty to shape without using force, application by hand | 1 | 10 min after combining the compounds | **4** |  |  |
| sil.2 | 1 | 1.718 | 2 | medium viscosity; application with manufacture-built device limits creation of adhesive drops <2 mm | 1 | 15 min after combining the compounds | **4** | 1 | **5** |
| sil.3 | 1 | 2.084 | 2 | medium viscosity; application by picking adhesive up with plastic stick tip limits creation of adhesive drops <2 mm | 1 | 15 min air curing | **4** |  |  |
| fire.4 | 1 | 1.716 | 0 | high viscosity; difficulty to shape without using force | 3 | 20 s blue light | **4** |  |  |
| cem.1 | 0 | 2.303 | 2 | medium viscosity; application by picking adhesive up with plastic stick tip limits creation of adhesive drops <2 mm | 1 | 20 min after combining the compounds | **3** | 2 | **5** |
| cem.2 | 0 | 2.195 | 2 | medium viscosity; application by picking adhesive up with plastic stick tip limits creation of adhesive drops <2 mm | 1 | 20 min after combining the compounds | **3** |  |  |
| cem.3 | 0 | 2.216 | 2 | medium viscosity; application by picking adhesive up with plastic stick tip limits creation of adhesive drops <2 mm | 1 | 20 min after combining the compounds | **3** |  |  |
| ha.2 | 0 | 2.809 | 0 | difficult to shape precisely due to high viscosity and insufficient application method; adhesive leaves streaks and sticks to manufacturer-built applicator, mixing components without air entrapment is challenging | 0 | 20 min after combining the compounds | **0** |  |  |

**Online supplement 3** Examples for the effect of spherical or flat application of adhesive unre.1 onto eight mouse crania in an ofMRI setting. Presented are coronal slices at the fiber insertion site (0 mm, yellow arrows) and directly adjacent to that (+ or – 0.5 mm). Images were acquired with a standard spin echo sequence (RARE, rapid acquisition with relaxation enhancement) as well as a gradient echo EPI (see text for scan parameters). Hyperintense areas dorsal to the cranium are agar patches that should minimize susceptibility effects at the air-tissue-interface. Adhesive patches are marked with white arrowheads and delineated by the dorsal hypointense area in the RARE images


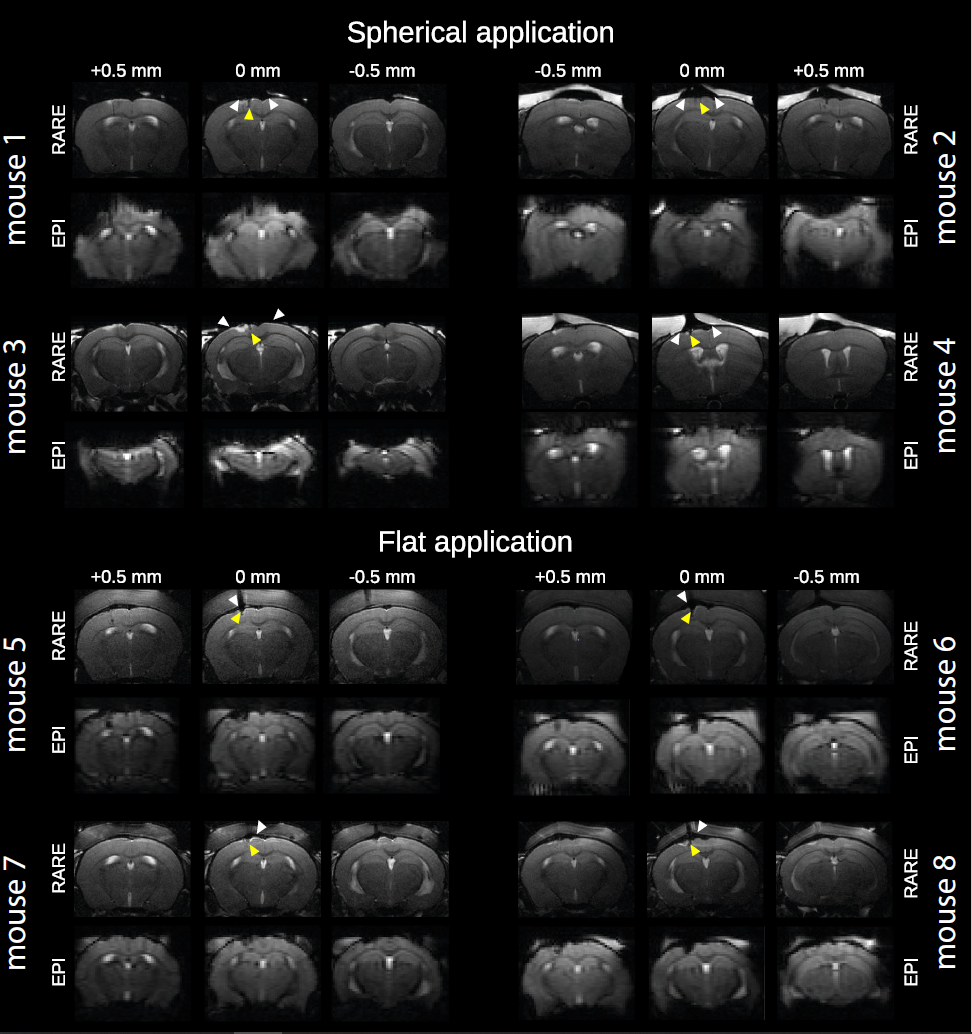

Supplement: Supplementary file 1 — Supplementary file1 (DOCX 696 kb) [file 10334_2024_1220_MOESM1_ESM.docx]
